# Modified oral cryotherapy is associated with lower oral mucositis pain in HSCT recipients: a retrospective study

**DOI:** 10.3389/fonc.2026.1704214

**Published:** 2026-04-01

**Authors:** Li-Zhu Huang, Mei-hua Chen, Xiao-fan Li, Yan Liu, Wei Lin, Ai-Ming Chen, Nai-Nong Li

**Affiliations:** 1 Fujian Institute of Hematology, Fujian Provincial Key Laboratory on Hematology, Fujian Medical University Union Hospital, Hematopoietic Stem Cell Transplantation Center Nursing Team, Fuzhou, Fujian, China; 2 Fujian Institute of Hematology, Fujian Provincial Key Laboratory on Hematology, Fujian Medical University Union Hospital, Fuzhou, Fujian, China

**Keywords:** HSCT, Kangfuxin solution, oral cryotherapy, oral mucositis, pain management

## Abstract

**Purpose:**

To assess modified oral cryotherapy (OC) for pain management in hematopoietic stem cell transplant (HSCT) recipients with oral mucositis (OM).

**Methods:**

A retrospective study included 361 HSCT patients (2023-2025) stratified into Conventional Nursing Practice (CNP) group (n=170) and optimized nursing protocol (ONP) group (n=191). The ONP protocol replaced saline ice slurry with Kangfuxin solution ice slurry and incorporated adjunctive bilateral cheek cryotherapy using ice packs. Outcomes compared OM severity and pain levels (Verbal Rating Scale).

**Results:**

Both groups showed comparable overall OM incidence (78.8% vs 78.0%, P = 0.851). The optimized protocol was associated with moderate-to-severe pain (Grade 2–3: 22.8% vs 39.6%, ARR = 16.8%, P = 0.004), with a 66% reduction in severe pain (Grade 3: 4.0% vs 11.9%). Multivariable analysis showed that age, alcohol use, and baseline oral pathology were independently associated with OM (all P < 0.05).

**Conclusion:**

Modified OC incorporating Kangfuxin ice slurry and localized cheek cryotherapy was associated with OM-related pain severity without reducing overall incidence, suggesting a clinically feasible and effective strategy for pain control in HSCT recipients.

## Background

Hematopoietic stem cell transplantation (HSCT) is an effective treatment for hematologic malignancies, genetic diseases, and certain solid tumors, with demonstrated favorable outcomes (1). However, high-dose conditioning regimens frequently lead to severe oral mucositis (OM), which occurs in 35–75% of autologous and 43.7–100% of allogeneic HSCT recipients (2). Clinical manifestations include mucosal erythema, ulceration, pain, and taste dysfunction, often resulting in dysphagia, nutritional deficiency, and increased infection risk, thereby compromising treatment adherence and transplant success (3). The WHO classifies OM into grades 0–IV; severe cases (grades III–IV) require liquid diets, parenteral nutrition, and opioid analgesia (4). Current management focuses on systematic oral care, antiseptic rinses, and cryotherapy to reduce mucosal injury and promote recovery (5).

Oral cryotherapy (OC) is internationally recommended to prevent chemotherapy-induced oral mucositis (OM) by constricting blood vessels and reducing mucosal damage (6). To enhance this effect, our center integrated Kangfuxin Solution—a traditional Chinese medicine extract from *Periplaneta americana*—into the cryotherapy protocol. Kangfuxin Solution contains bioactive components (e.g., amino acids, peptides, mucopolysaccharides) demonstrated to promote mucosal repair and exert anti-inflammatory and analgesic effects (7–9). This combination was implemented to synergize the physical vasoconstrictive effect of cryotherapy with the pharmacological actions of Kangfuxin, providing a rationale for our modified approach to OM symptom management.

Our center has developed a modified OC protocol that innovatively replaces saline-prepared ice with Kangfuxin solution and combines it with localized hypothermic patch application on the cheek area. This study employed a retrospective analysis to validate the analgesic efficacy of the optimized protocol in OM-related pain management, and to identify potential risk factors for OM development, aiming to establish an evidence-based rationale for personalized care strategies.

## Methods

### Patient selection

This study retrospectively analyzed clinical data from patients undergoing HSCT at Fujian Medical University Union Hospital between January 2023 and January 2025.Inclusion criteria: (1) Age >18 years; (2) The patient met the indications for HSCT and successfully underwent the treatment at our institution. Exclusion criteria comprised: (1) History of malignancy or concurrent malignancies in other anatomical sites; (2) Pre-existing OM prior to chemotherapy; (3) The patient declined or was unable to comply with OC; (4) complicated by varicella-zoster virus infection or with active infection; (5) The data were incomplete. All procedures in this study were reviewed and approved by the Ethics Committee of Fujian Medical University Union Hospital. The study strictly adhered to national ethical regulations and complied with the ethical standards outlined in the 1964 Declaration of Helsinki and its subsequent amendments.

A total of 361 patients were included based on the predefined inclusion and exclusion criteria. Since January 2024, an optimized OM protocol has been implemented at our center. All patients received conventional nursing care from January to December 2023, therefore, patients were stratified into two groups based on temporal criteria. Patients were allocated to the Conventional Nursing Practice (CNP) group (n=170) and the optimized nursing protocol (ONP) group.

### Conventional nursing practice

The institutional nursing protocol mandates OC using slushy saline solution initiated at chemotherapy commencement. To prevent oral frostbite, moderate activation of the tongue and masticatory muscles was recommended to enhance local blood circulation, the head was gently tilted backward to facilitate complete filling of the oral cavity and ensure the substance reached the pharyngeal region, the patient was instructed to repeatedly perform sucking and cheek puffing maneuvers until the ice chips were completely melted, followed by expulsion of the ice water. Patients were instructed to continue gargling with the same method until the entire prepared dose was consumed, with each session lasting approximately 10 minutes, repeated three times daily until discharge. The saline ice slush was standardized and prepared by healthcare professionals, The standardized 100 mL PVC soft bags containing sodium chloride injection for intravenous infusion were frozen in a freezer for 2.5 hours as the experimental protocol, the standardized 100 mL PVC soft-packaged 0.9% sodium chloride infusion bag was placed in a freezer set to approximately −20 °C for 2.5 hours to prepare a slushy saline mouthwash.

### Optimized nursing protocol

Since January 2024, an optimized cryotherapy protocol has been implemented for patients in the ONP group in this study. The protocol includes two key improvements: replacing the traditional saline ice slurry with Kangfuxin solution ice slurry, and innovatively adopting ethanol ice balls for bilateral cheek transcutaneous cryotherapy. The preparation process of Kangfuxin ice slurry remains consistent with conventional methods to ensure operational consistency. This modification is theoretically grounded in the pharmacological properties of Kangfuxin solution, which is rich in bioactive components such as amino acids, peptides, and polysaccharides. Its pharmacological characteristics can synergize with cryotherapy: while cold-induced vasoconstriction reduces local blood flow and chemotherapy drug exposure, Kangfuxin solution actively promotes mucosal repair and regeneration, while exerting anti-inflammatory and analgesic effects.

For cheek cryotherapy, specially formulated ethanol ice balls were used: A mixture of 50ml sterile saline (0.9% NaCl) and 50ml 75% medical ethanol was sealed in infusion bag and frozen at -20 °C for 4–6 hours. The precise ratio of ethanol concentration and freezing time ensures the mixture forms an ideal slushy state rather than a completely solid ice block. This physical state provides improved plasticity and extensibility, allowing better conformity to facial anatomical contours, thereby offering a larger effective cold compress contact area while avoiding excessive local pressure.

During the treatment process, these specially prepared ethanol ice balls were wrapped in medical gauze and applied simultaneously to both cheeks during each 10-minute oral cryotherapy session. This method was designed to improve patient tolerance and compliance by reducing direct cold stimulation in the oral cavity, while enhancing the low-temperature protection effect on the deep buccal mucosal area through sustained surface cooling of the cheek area. This combined treatment approach integrates dual strategies of physical cooling and pharmacological therapy, providing a practical approach for pain management in oral mucositis, and allowing patients to use auxiliary cheek cryotherapy as needed during episodes of breakthrough pain.

### Definition

In this study, smoking history was defined according to the WHO dichotomous criteria for tobacco use, incorporating both self-reported data and medical records (10).Participants were categorized into two groups: those with a smoking history (defined as having regularly smoked ≥1 cigarette daily for ≥1 year) and non-smokers (those who did not meet this criterion or were lifelong non-smokers).The definition of alcohol consumption history is established according to the (WHO) Global Status Report on Alcohol and Health (11).Participants were stratified by pre-chemotherapy alcohol consumption history (≥1 standard drink/week for ≥6 months vs non-drinkers/lower intake). One standard drink contains 14 g of pure ethanol, equivalent to 350 mL of beer (approximately 3-8% alcohol by volume) or 45 mL of Chinese Baijiu (typically 38-53% alcohol by volume).

Patients were categorized into two groups based on their prechemotherapy oral health status: favorable and poor. Good oral health status is defined as the absence of active dental caries (untreated carious surfaces = 0) and periodontal disease (gingival bleeding index ≤10%), and had no dentures or dental prostheses. Poor oral status was defined as the presence of ≥1 untreated decayed teeth according to the WHO caries assessment criteria (12), or presenting with periodontal disease (bleeding on probing-positive sites ≥30%) and/or denture-related mucosal lesions.

The diagnosis of OM was assessed according to the WHO grading criteria for OM (13). Grade 0: No detectable alterations of the oral mucosa; Grade I: Erythema and/or pain in the oral mucosa with 1–2 small ulcers (<1 cm diameter), maintaining ability to tolerate a regular diet; Grade II: Single large ulcer (>1 cm) with severe pain requiring semi-solid diet; Grade III: Two large ulcers (>1 cm) requiring liquid diet; Grade IV: Confluent ulcerative plaques (>1 cm) with incapacitating pain precluding oral intake.

### Observed parameters

#### Subjective indicators

Oral pain was assessed each morning by uniformly trained and certified bedside nurses using standardized scales, with the highest pain intensity from the previous day recorded as the study’s pain score. The assessment was conducted using the Verbal Rating Scale (VRS) (14), which categorizes pain intensity into four grades based on patient-reported symptoms, Grade 0: No pain; Grade I (Mild): Tolerable pain with normal daily activities and no sleep disturbance; Grade II (Moderate): Significant pain requiring analgesic medication, accompanied by sleep interference; Grade III (Severe): Intolerable pain necessitating analgesic use, severe sleep disruption, and potential autonomic nervous system dysfunction or forced positioning.

#### Objective indicators

Objective indicators were used to complement the verification of pain severity and its demand for treatment, primarily including two types of analgesic measures taken due to oral pain. The first was the use of systemic analgesics, referring to systemic pain medications (including opioid patches, injectable formulations, etc.) administered for intolerable pain. The second was the use of lidocaine gel, which was given as a mouth rinse for local analgesia due to patient oral pain. We separately calculated the proportion of patients using lidocaine gel (usage rate) in each group and, among those who used the gel, calculated the mean daily application frequency per patient to more accurately reflect the frequency and intensity of pain management.

### Statistical method

In data analysis, Categorical variables were presented as frequency (percentage), while continuous variables were described using mean ± standard deviation (SD) for normally distributed data or median (interquartile range, IQR) for non-normally distributed data. The intergroup comparisons were conducted using the following statistical methods: Categorical variables were analyzed using the Chi-square test or Fisher’s exact test, as appropriate. For continuous variables, the independent samples t-test was applied to normally distributed data, whereas the Mann-Whitney U test was utilized for non-normally distributed data. In this study, binary logistic regression analysis was employed to identify the independent risk factors associated with OM. Potential predictors were initially screened using univariate logistic regression (significance threshold: P<0.05). Variables meeting this criterion were subsequently incorporated into a multivariable logistic regression model to identify independent risk factors. All statistical analyses were performed with SPSS Statistics software (version 26.0 for Windows; IBM Corp., Armonk, NY, USA). Statistical analyses were performed using two-tailed tests, and a P value of <0.05 was deemed statistically significant.

## Result

### Patient baseline characteristics

A total of 361 patients who underwent hematopoietic stem cell transplantation (HSCT) were ultimately enrolled in this study. Based on the time of enrollment, 170 patients (47.09%) were assigned to the Conventional Nursing Practice (CNP) group and 191 patients (52.91%) to the Optimized Nursing Protocol (ONP) group. No statistically significant differences were observed between the two groups regarding age, sex, body mass index (BMI), history of alcohol consumption, smoking history, baseline oral health status, disease type, transplantation method, or incidence of acute graft-versus-host disease (aGVHD) (all *P* > 0.05), indicating comparability between the groups (**Table 1**).

**Table 1:** Demographic and clinical characteristics with group comparisons.

Variables	Total (n = 361)	CNP group (n = 170)	ONP group (n = 191)	*P*
Age, M (Q_1_, Q_3_)	44.00 (25.00, 55.00)	43.00 (23.00, 55.00)	45.00 (28.50, 56.00)	0.420
BMI, M (Q_1_, Q_3_)	20.31 (17.42, 23.44)	20.52 (17.14, 23.09)	20.10 (17.64, 23.76)	0.560
Sex, n(%)				0.133
F	176 (48.75)	90 (52.94)	86 (45.03)	
M	185 (51.25)	80 (47.06)	105 (54.97)	
Drink, n(%)				0.773
No	265 (73.41)	126 (74.12)	139 (72.77)	
Yes	96 (26.59)	44 (25.88)	52 (27.23)	
Smoking, n(%)				0.839
No	244 (67.59)	114 (67.06)	130 (68.06)	
Yes	117 (32.41)	56 (32.94)	61 (31.94)	
Oral Condition, n(%)				0.901
Healthy	275 (76.18)	129 (75.88)	146 (76.44)	
Pathological	86 (23.82)	41 (24.12)	45 (23.56)	
OM grade, n(%)				0.996
0	154 (42.66)	71 (41.76)	83 (43.46)	
1	94 (26.04)	45 (26.47)	49 (25.65)	
2	66 (18.28)	31 (18.24)	35 (18.32)	
3	32 (8.86)	16 (9.41)	16 (8.38)	
4	15 (4.16)	7 (4.12)	8 (4.19)	
Transplantation Method, n(%)				0.281
Auto-HSCT	125 (34.63)	54 (31.76)	71 (37.17)	
Allo-HSCT	236 (65.37)	116 (68.24)	120 (62.83)	
Disease category, n(%)				0.832
Aplastic Anemia	58 (16.07)	27 (15.88)	31 (16.23)	
Leukemia	175 (48.48)	81 (47.65)	94 (49.21)	
Lymphoma	57 (15.79)	30 (17.65)	27 (14.14)	
Multiple Myeloma	71 (19.67)	32 (18.82)	39 (20.42)	
Oral Mucositis, n(%)				0.851
No	78 (21.61)	36 (21.18)	42 (21.99)	
Yes	283 (78.39)	134 (78.82)	149 (78.01)	
Hospital Stay	41.28±12.41	41.7±10.87	40.9±13.65	0.542
GVHD (+), n(%)	181	87	94	0.709

ONP Group: optimized nursing protocol group; CNP Group, Conventional Nursing Practice group; GVHD, Graft-versus-Host Disease.

### Oral mucositis incidence and pain assessment

**Figure 1:**
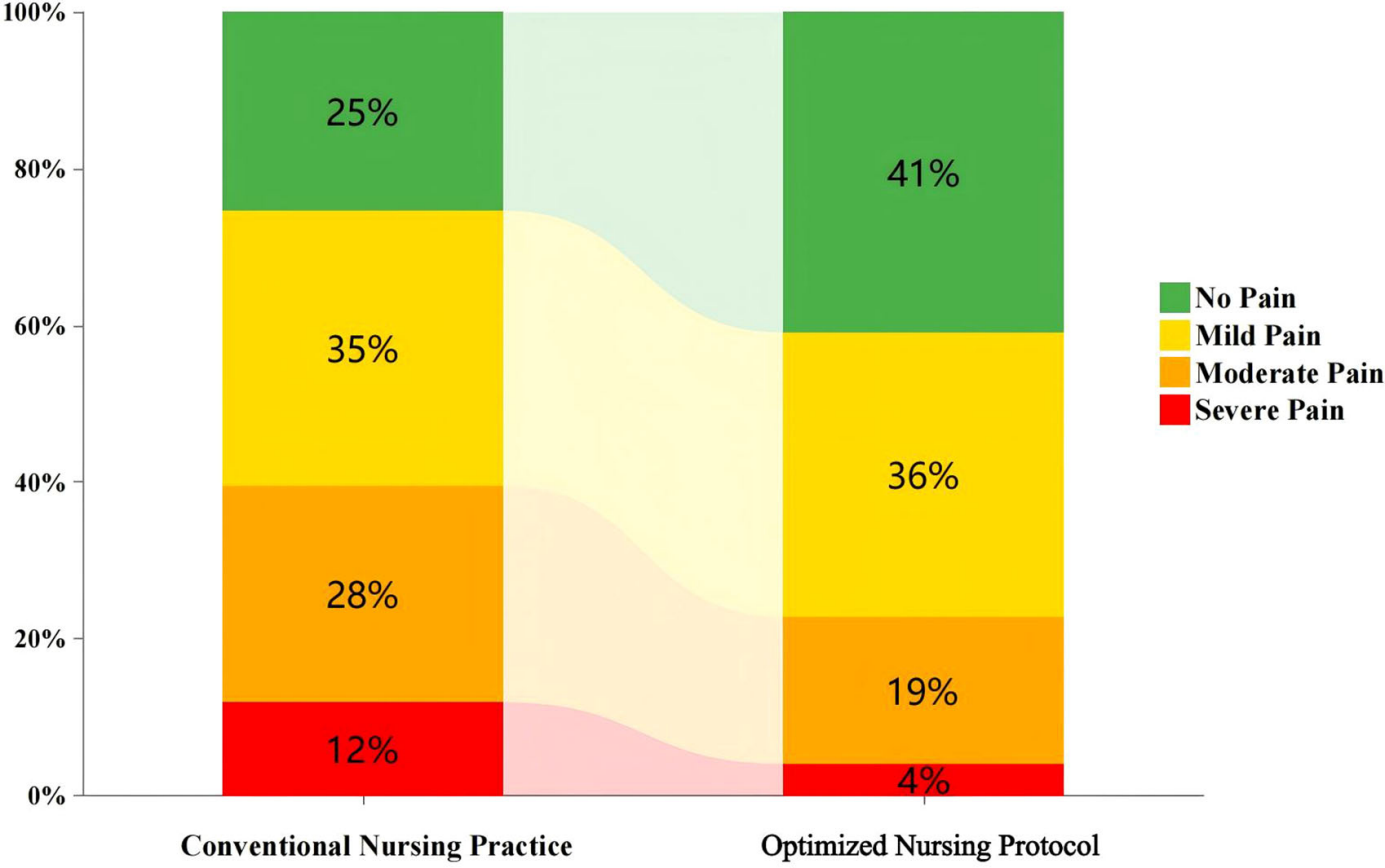
Comparative distribution of pain severity between optimized and improved nursing protocols.

The overall incidence of oral mucositis (OM) was similar between the two groups, with no statistical difference (78.82% in CNP vs. 78.01% in ONP, *P* = 0.851). However, a significant difference was found in the distribution of pain severity (*P* = 0.010). Specifically, the ONP group had a higher proportion of patients with no pain (Grade 0: 53.93% vs. 41.18%) and a lower proportion of patients with moderate-to-severe pain (Grades 2 and 3 combined: 17.80% vs. 31.17%) compared to the CNP group (**Figure 1**; **Table 2**). Regarding objective treatment measures, the usage rate of lidocaine gel was significantly lower in the ONP group than in the CNP group (20.94% vs. 33.53%, *P* = 0.007), and the mean daily application frequency per patient was also lower (2.10 ± 1.04 times vs. 2.75 ± 0.9 times, *P* < 0.001). No significant difference was observed in the usage rate of systemic analgesics between the groups (35.08% vs. 43.53%, *P* = 0.100). Notably, these objective findings were consistent with the subjective VRS distribution, supporting the validity of the observed between-group differences in pain severity.

**Table 2:** Pain assessment for oral mucositis after hematopoietic stem cell transplantation.

Variables	Item	Category	CNP group	ONP group	p
Subjective Evaluation	Pain Grade, n(%)	0	70 (41.18)	103 (53.93)	0.010
		1	47 (27.65)	54 (28.27)	
		2	37 (21.76)	28 (14.66)	
		3	16 (9.41)	6 (3.14)	
Objective Evaluation	Systemic Analgesics, n(%)	No	96(56.47)	124 (64.92)	0.100
		Yes	74(43.53)	67 (35.08)	
	Lidocaine Gel, n(%)	No	113(66.47)	151 (79.06)	0.007
		Yes	57(33.53)	40 (20.94)	
	Mean Application Frequency of Lidocaine Gel per Patient		2.75±0.9	2.10±1.04	<0.001

### Risk factor analysis for oral mucositis

**Figure 2:**
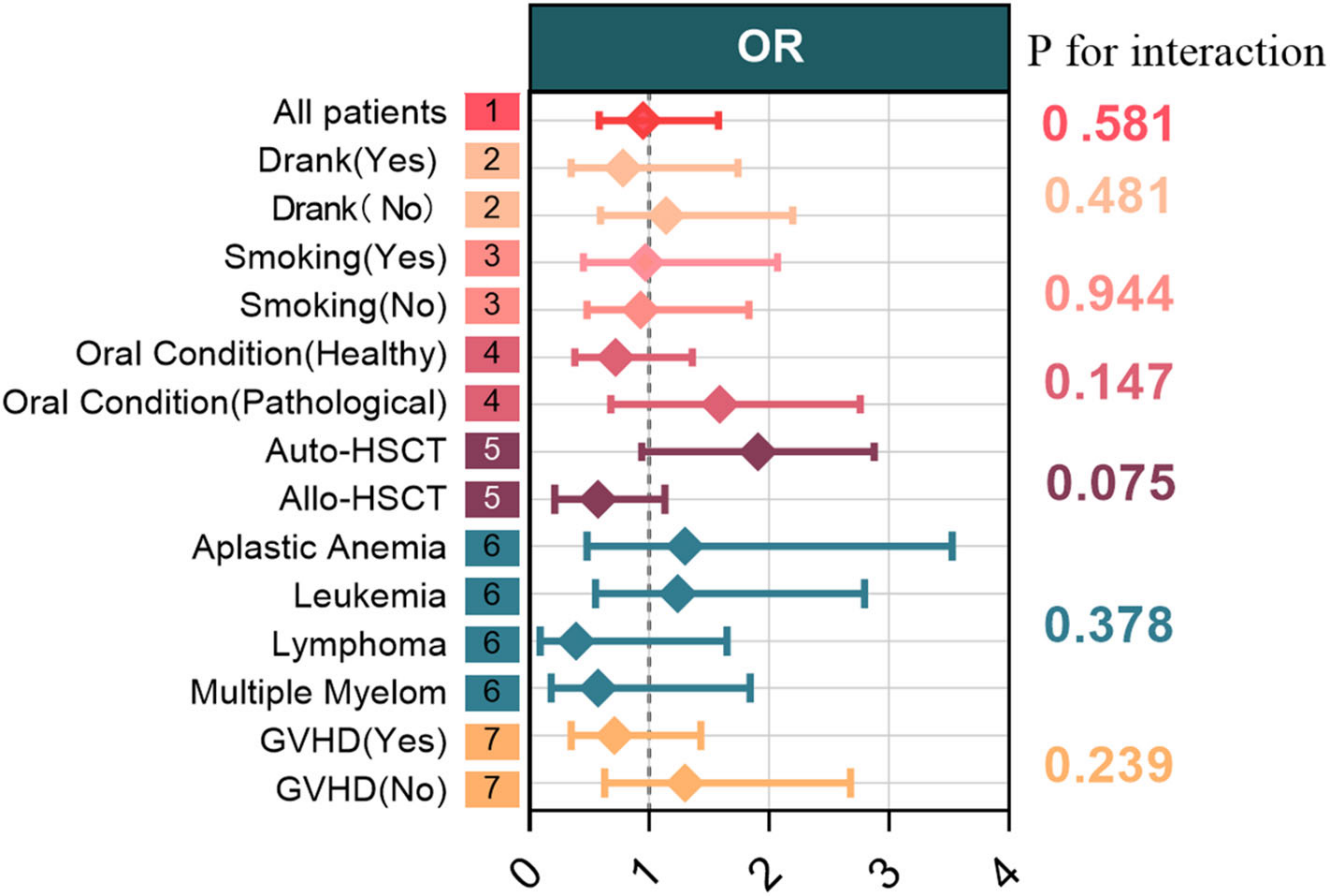
Forest plot of subgroup analyses. The left side shows the subgroup categories. The diamonds in the center represent odds ratios (ORs), and the horizontal lines represent the 95% confidence intervals (CIs). The vertical line in the center is the null line (OR = 1). If a confidence interval crosses the null line, it indicates that the difference is not statistically significant. The values on the right are P-values for interaction.

Multivariable logistic regression analysis identified advanced age (OR = 1.02, 95% CI: 1.01–1.04, *P* = 0.017), a history of alcohol consumption (OR = 3.48, 95% CI: 1.64–7.38, *P* = 0.001), and the presence of baseline oral pathological conditions (OR = 3.32, 95% CI: 1.51–7.31, *P* = 0.003) were independently associated with OM (**Table 3**). Subsequent subgroup analyses (**Figure 2**) demonstrated that the associations of these risk factors remained consistent across patient subgroups with different clinical characteristics (e.g., different transplantation methods, disease types), with all interaction *P*-values greater than 0.05, further supporting their generalizability as risk factors.

**Table 3:** Risk factor analysis for oral mucositis in HSCT patients: univariate and multivariate models.

Variables	Univariable regression analysis					Multivariable regression analysis				
	β	S.E	Z	*P*	OR (95%CI)	β	S.E	Z	*P*	OR (95%CI)
Age	0.02	0.01	2.14	0.032	1.02 (1.01 ~ 1.04)	0.02	0.01	2.38	0.017	1.02 (1.01 ~ 1.04)
BMI	0.02	0.03	0.62	0.537	1.02 (0.96 ~ 1.09)					
Sex										
F					1.00 (Reference)					
M	-0.20	0.26	-0.77	0.439	0.82 (0.50 ~ 1.36)					
Drink										
No					1.00 (Reference)					1.00 (Reference)
Yes	1.22	0.38	3.25	0.001	3.40 (1.63 ~ 7.13)	1.25	0.38	3.25	0.001	3.48 (1.64 ~ 7.38)
Smoking										
No					1.00 (Reference)					
Yes	0.86	0.31	2.75	0.006	2.37 (1.28 ~ 4.37)					
Oral condition										
Healthy					1.00 (Reference)					1.00 (Reference)
Pathological	1.20	0.40	3.04	0.002	3.33 (1.53 ~ 7.24)	1.20	0.40	2.98	0.003	3.32 (1.51 ~ 7.31)
Transplantation Method										
Auto-HSCT					1.00 (Reference)					
Allo-HSCT	-0.30	0.28	-1.08	0.282	0.74 (0.43 ~ 1.28)					
Disease category										
0					1.00 (Reference)					
1	-0.22	0.39	-0.55	0.581	0.80 (0.37 ~ 1.74)					
2	-0.77	0.43	-1.78	0.076	0.46 (0.20 ~ 1.08)					
3	-0.02	0.49	-0.04	0.966	0.98 (0.37 ~ 2.57)					
Group										
CNP Group					1.00 (Reference)					
ONP Group	-0.05	0.26	-0.19	0.851	0.95 (0.58 ~ 1.58)					
GVHD										
No					1.00 (Reference)					
Yes	-0.08	0.21	-0.36	0.719	0.93 (0.61 ~ 1.40)					

## Discussion

This retrospective cohort analysis systematically evaluated the clinical value of a modified oral cryotherapy protocol within the comprehensive management of oral mucositis (OM) in patients undergoing hematopoietic stem cell transplantation (HSCT). Our primary findings reveal a pivotal and intriguing clinical phenomenon: compared to the conventional nursing protocol (CNP), the optimized nursing protocol (ONP), while failing to significantly reduce the overall incidence of OM (78.01% vs 78.82%, *P* = 0.851), yielded the most patient-centric was associated with lower pain severity. This was particularly evidenced by a lower proportion of severe pain (4.0% vs 11.9%). This result suggests that the core effect of the modified cryotherapy may lie in modulating the clinical presentation and symptom burden of mucositis, rather than preventing its initial occurrence.

The superior efficacy of the optimized protocol in pain management confirms the success of our rationale based on a synergistic mechanism. International consensus guidelines endorse oral cryotherapy as an effective intervention for preventing OM in HSCT recipients (6), primarily through cold-induced vasoconstriction to reduce mucosal damage (6). Our modified protocol innovatively integrates Kangfuxin Solution, a traditional Chinese medicine extract with demonstrated pharmacological activities. Kangfuxin Solution, derived from *Periplaneta americana*, is rich in bioactive components such as polyhydroxy alcohols, amino acids, peptides, and mucopolysaccharides, which have been shown to promote mucosal repair and exert anti-inflammatory and analgesic effects (9). The underlying analgesic mechanisms are multifaceted: polyols can alleviate ischemia- and inflammation-mediated pain by improving local microcirculation and promoting the sloughing of necrotic tissue (9); mucopolysaccharides can activate macrophages and natural killer (NK) cells, stimulating the secretion of anti-inflammatory mediators to suppress inflammatory responses and indirectly relieve pain (9, 15); furthermore, the solution promotes cell proliferation and migration, thereby shortening pain duration through accelerated tissue repair (16). As the Kangfuxin ice slurry melts in the oral cavity, its active ingredients directly act on the damaged mucosa, superimposing pharmacological anti-inflammatory and reparative actions onto the physical analgesia of cryotherapy. Additionally, our novel adjunctive bilateral cheek cryotherapy using ethanol ice balls not only enhances the low-temperature protection of the deep buccal mucosal area via sustained surface cooling but also crucially improves treatment tolerance. This addresses a significant limitation noted in previous research, where patient intolerance to intraoral cryostimulation often led to premature termination of therapy (17). This synergy between physical cooling and pharmacological therapy forms the theoretical basis for our protocol’s effectiveness in controlling pain and reducing the reliance on topical anesthetics like lidocaine gel (ONP group vs CNP group: 20.94% vs 33.53%, *P* = 0.007). Notably, the ONP was implemented as a bundled, two-component protocol; thus, the observed association with lower pain severity cannot be attributed to Kangfuxin or cheek cooling alone. It remains possible that expanded extra-oral cooling (surface area/duration) contributed substantially, which is relevant for reproducibility and cost-effectiveness.

The inability of this approach to reduce the overall incidence of OM is a critical point for discussion. The initiation of OM is largely attributable to the direct cytotoxic damage of conditioning chemotherapies to the basal epithelial cells, a process that is inherently difficult to completely abrogate. Therefore, the comparable overall incidence between the two groups may reflect a ceiling effect of cryotherapy at the primary preventive “front line.” This finding aligns with the focus of the Cochrane review by Riley et al. (17) which primarily affirmed the efficacy of cryotherapy in reducing OM *incidence*, with less emphasis on its role in *pain management*. However, this differential outcome underscores the value of our optimized protocol on the “second front”: it likely alters the disease course and character of OM. In other words, while modified cryotherapy may not prevent the emergence of mucosal erythema or ulceration, it may help attenuate the local inflammatory cascade, may be associated with less tissue edema, and may be linked to faster repair processes around ulcers. This transforms a “painful, highly inflammatory” severe OM into a “symptom-controlled, manageable” mild condition. This perfectly explains the significant reduction in the proportion of patients with moderate-to-severe pain in the ONP group. Consequently, our findings indicate that the primary clinical value of the modified cryotherapy strategy lies in its “disease-modifying” effect, rather than mere prevention.

Beyond evaluating the intervention, identifying high-risk populations is crucial for optimizing resource allocation. Our multivariable analysis established advanced age (OR = 1.02), a history of alcohol consumption (OR = 3.48), and the presence of baseline oral pathological conditions (OR = 3.32) as independent risk factors for OM development, with these associations remaining consistent across patient subgroups. Older age may confer challenges such as reduced epithelial growth factor production, diminished proliferative capacity of oral epithelial cells, and salivary gland dysfunction leading to microbial dysbiosis (18). A history of alcohol consumption, even at moderate levels as defined by WHO criteria (19), may increase OM risk by suppressing neutrophil function and reducing salivary secretion (20). Pre-existing oral pathologies, such as untreated dental caries and periodontal disease (defined according to WHO oral health survey methods (12)), provide a “breeding ground” for OM, echoing the findings of Skallsjö et al. (21) These findings suggest for a stratified preventive strategy prior to HSCT. While the modified cryotherapy may be feasible for routine implementation for all patients to control symptoms, high-risk patients may benefit from intensified pre-transplant interventions, such as mandatory comprehensive oral health assessment and treatment, and systematic alcohol cessation counseling.

### Limitation

This study has several limitations. Because groups were defined by calendar time, residual time-related confounding cannot be fully excluded, although conditioning regimens and routine supportive-care/analgesic practices at our center were institutionally stable during the study period. In addition, the ONP was implemented as a bundled, two-component protocol (Kangfuxin substitution plus adjunctive cheek cryotherapy), so we could not isolate the individual contribution of each component to pain outcomes; it is possible that expanded extra-oral cooling alone contributed substantially, which has implications for reproducibility and cost-effectiveness. The retrospective, single-center design may also involve unmeasured confounding despite multivariable adjustment, and pain assessment remains subjective with potential variability in cryotherapy adherence/application; future prospective studies with blinded assessment, objective biomarkers, and component-separation (e.g., factorial) designs are warranted. Finally, the lack of long-term follow-up on quality of life and oral function limits a comprehensive evaluation of overall benefit; therefore, findings should be interpreted as associations rather than causal effects.

## Conclusion

The findings of this study identified advanced age, alcohol consumption history, and oral pathological status as independent risk factors, highlighting the clinical imperative to prioritize oral health management in elderly patients and individuals with alcohol use history for potential risk mitigation. Although the ONP did not significantly reduce the overall incidence of OM, it demonstrated a 66% reduction in moderate-to-severe pain intensity. Notably, the protocol’s operational simplicity supports its feasibility for widespread clinical implementation.

## Data Availability

The original contributions presented in the study are included in the article/supplementary material. Further inquiries can be directed to the corresponding author.
